# The Neural Sulcus of the Cervical Vertebrae: A Review of Its Anatomy and Surgical Perspectives

**DOI:** 10.7759/cureus.6693

**Published:** 2020-01-18

**Authors:** Neil Joshi, Neil Klinger, Dia R Halalmeh, R. Shane Tubbs, Marc D Moisi

**Affiliations:** 1 Neurological Surgery, Banner University Medical Center, Phoenix, USA; 2 Neurological Surgery, Wayne State University School of Medicine, Detroit, USA; 3 Neurological Surgery, Detroit Medical Center, Detroit, USA; 4 Clinical Anatomy, Seattle Science Foundation, Seattle, USA

**Keywords:** neural sulcus, cervical, posterior, screw, anatomy, surgical

## Abstract

The neural sulcus is a bony channel that spans the transverse process in the subaxial cervical spine. It is located between the anterior and posterior tubercles on either side of the transverse foramen, housing the spinal nerve as it passes through the intervertebral foramina. Although numerous studies have evaluated the anatomy of the cervical spine, very little data on detailed anatomy of the neural sulcus and its implication in cervical spine surgery exist. Here, we review the anatomy of the neural sulcus and surgical considerations. The neural sulcus has important surgical implications, and knowledge of its anatomy is important in considering and planning posterior cervical segmented instrumentation. This increases the ability of the neurosurgeon to choose the best suitable surgical approach to the subaxial cervical spine, allowing good outcomes for the patient.

## Introduction and background

The typical vertebral body consists of a body and a posterior vertebral arch with several processes for muscular and articular attachments. The cervical vertebrae are characterized by the presence of round foramen, perforating each transverse process. The foramen transversarium in cervical vertebrae other than the atlas is bounded by the anterior tubercle anteriorly and by the posterior tubercle posteriorly [1,2]. The neural sulcus is an osseous channel of the transverse process of the subaxial cervical vertebrae that imparts added protection to the cervical nerves as they exit the spinal canal. Additionally, it allows for a direct unimpeded pathway through which the vertebral artery passes between the adjacent transverse foramina [1-3]. As only very scant reports have looked at this anatomical structure in detail, the following review was performed.

## Review

Anatomy

**Figure 1:**
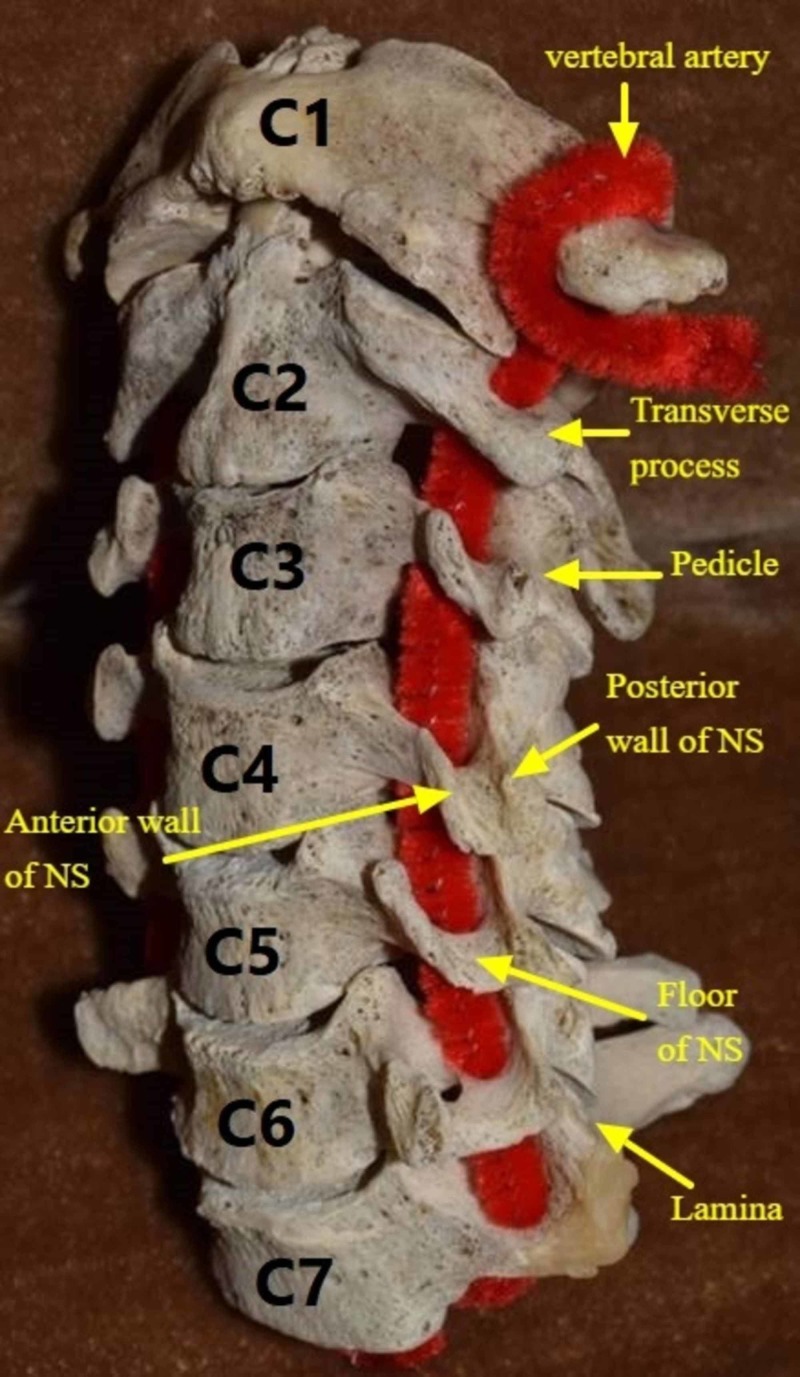
Neural sulcus boundaries. NS: Neural Sulcus

**Figure 2:**
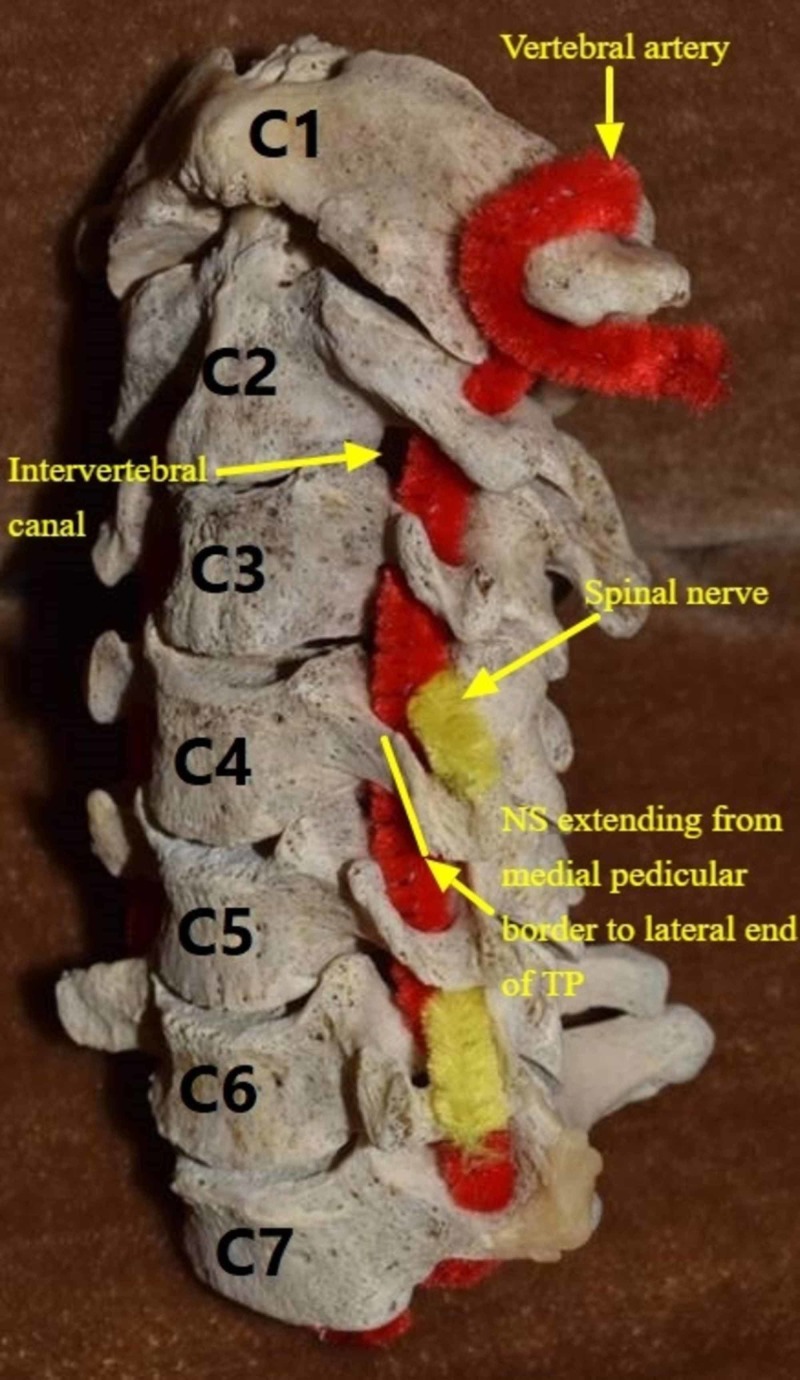
The neural sulcus and related neural elements. NS: Neural Sulcus; TP: Transverse Process

In the subaxial cervical spine, the dorsal and ventral nerve roots exit the vertebral canal via the intervertebral foramen. They continue their course laterally by way of the neural sulcus, which extends from the medial border of the pedicle to the lateral end of the transverse process and associated costal process [1-3]. It is bound by the anterior and posterior tubercles (Figure 1). In the medial segment of the canal, nerve roots are located superior to the pedicle of the adjacent vertebra, anteromedial to the medial portion of the articular process, and posterolateral to the uncinate process. The cervical nerve roots unite more laterally, forming the spinal nerve before splitting to form the dorsal and ventral primary rami. These neural elements are situated within the neural sulcus and lie posterior to the vertebral artery (Figure 2). Laterally, the ventral ramus continues to lie within the neural sulcus and is bounded anteriorly and posteriorly by the ridges of the true transverse and costal processes and associated tubercles, respectively [1,2].

**Figure 3:**
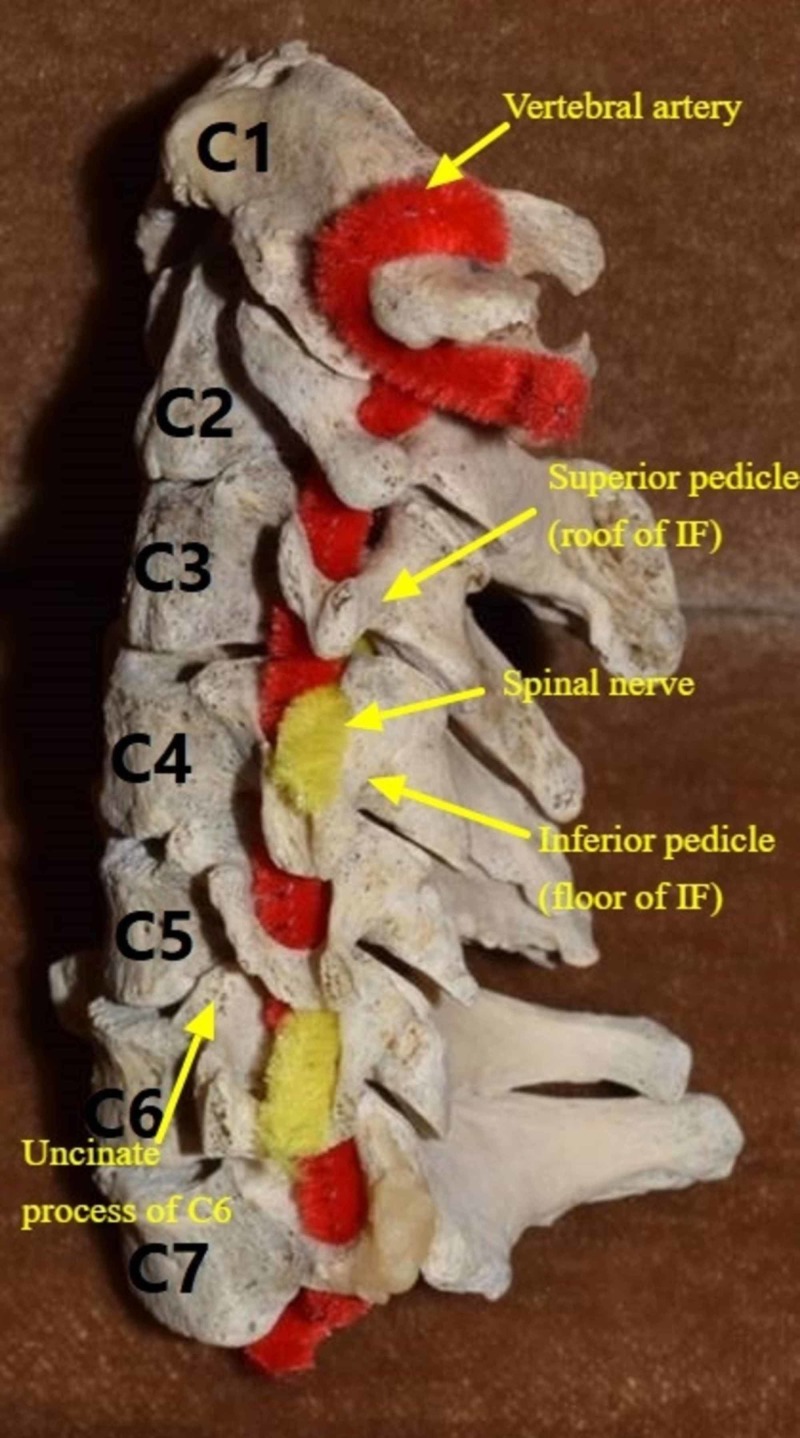
The medial region of the neural sulcus IF: Intervertebral Foramen

The uncotransversoarticular complex, described in previous investigations, as the grouping of the uncinate and transverse processes and the pedicle and articular facet of each cervical vertebra, is significant in the patency of the neural sulcus along its full path. The neural sulcus extends from the vertebral canal to the tip of the transverse process with an average length of 1.5-2.0 cm [2]. It can be divided into two portions: a “radicular portion” (medial) which houses the spinal nerve and a “portion of the anterior ramus of the transverse process” (lateral). The radicular portion forms much of the neural sulcus, measuring 1 cm in length [2]. A different classification scheme was proposed by Ebrahim et al., who performed a cadaveric study that quantified the neural sulcus [1]. For this purpose, the neural sulcus was divided into three zones according to the different subjacent anatomical structures found along its length: a medial zone situated superior to the pedicle, a middle zone posterior to the vertebral artery, and a lateral zone. The medial zone is thought to be more important in radiculopathy than the middle and lateral zones [4]. This is because the adjacent superior and inferior pedicles, anterior uncinate process, and posterior superior facet create a tightly bound unit by intimately surrounding the medial region of the neural sulcus (Figure 3), which is highly susceptible to hypertrophy and spondylosis [1]. The width of the medial zone was found to be largest at C3, and no significant gender differences were identified. Similarly, the depths of the medial zones were found to be largest at C3. Interestingly, the average angle between the midsagittal plane and the takeoff of the neural sulcus was 49-50.5 degrees from C3-6, and this increased sharply to 56 degrees at C7 [1].

Surgical Relevance

Cervical radiculopathy is a debilitating condition, with an annual age-adjusted incidence rate of 83.2 per 100,000 [5]. Diagnosis typically includes physical exam findings, diagnostic imaging studies, and electromyography. The management of cervical radiculopathy can include conservative therapies (e.g. physical therapy, superficial heat, cervical traction, epidural nerve root injections) or surgical intervention [6]. Of particular interest to this review is determining how the neural sulcus impacts various surgical approaches to the subaxial cervical spine. More specifically, posterior cervical fusion techniques that require screw placement are examined. In the subaxial cervical spine, segmented instrumentation after decompression is required when there is instability or when postoperative kyphosis needs to be prevented. This can be accomplished in general by placement of screws for the fusion construct in the lateral mass or less commonly the pedicle. The goal of these procedures is to hold the spine rigid at these levels for arthrodesis to occur.

Lateral mass screw fixation was first described in 1964 and has undergone modifications since then to decrease complications [7-11]. Many methods are described that detail the screw entry point and trajectory. Using the Roy-Camille method, the screw is placed in the center of the lateral mass, bounded by the end of the facet laterally, the edges of the articular processes cranially and caudally, and the end of the lamina medially. A trajectory of 10 degrees lateral and zero degrees vertical angulation is used [7]. The entry point is different in the Magerl technique, where the screw is placed 2-3 mm medial and cephalad from the center of the lateral mass. The screw trajectory is then modified with 25 degrees lateral angulation and vertical angulation in line with the articular facet [12]. This trajectory allows for longer screw placement, and is associated with greater resistance to pullout [13].

Improperly sized and poorly placed lateral mass screws can cause iatrogenic injury to cervical nerve roots [1]. The intervertebral foramen lies anteromedially to the entry point of screws in the lateral mass, and screws that are too long or improperly angulated can pierce the cortex and possibly injure the exiting neural tissues. In addition, the transverse foramina that house the vertebral arteries are in the transverse process ventral and slightly lateral to the screw entry point. This drives rationale for lateral angulation of screws to mitigate risk to these structures. However, the neural elements continue laterally through the neural sulcus along the transverse process. Placing posterior transpedicular screws entails an even greater risk of disrupting the cervical nerve root by misplaced, misdirected, or improperly measured screws [1].

Fortunately, the neural sulcus supports the subaxial cervical nerve root and prevents surgical injury in several ways. First, it serves to secure the nerve during surgical manipulation, thereby mitigating long-term sequelae from iatrogenic injury [[1,2,14]. In addition to anchoring the subaxial cervical nerve root by fixing the exiting subaxial spinal nerve, the posterior wall of the neural sulcus directly protects the spinal nerve from penetration by misplaced lateral mass screws.Therefore, the neural sulcus directly shields each exiting subaxial cervical spinal nerve from screw penetration during surgery. The existence of the neural sulcus also clears the transverse foraminal and interforaminal pathways of the vertebral artery by forcing the intervertebral foramina to occupy a more anteromedial position [15].

## Conclusions

The neural sulcus facilitates the compact nature of the subaxial cervical spine by preserving neurovascular integrity. A thorough understanding of its anatomy and relationships to the surrounding area is essential during cervical spine surgery. This provides a safeguard against unwarranted damage to adjacent neural and vascular structures.
